# Splenectomy at early stage of autoimmune arthritis delayed inflammatory response and reduced joint deterioration in mice

**DOI:** 10.1093/cei/uxae013

**Published:** 2024-02-13

**Authors:** Esam Khanfar, Katalin Olasz, Szonja Gál, Erzsébet Gajdócsi, Béla Kajtár, Tamás Kiss, Péter Balogh, Timea Berki, Ferenc Boldizsár

**Affiliations:** Department of Immunology and Biotechnology, Medical School, University of Pecs, Pécs, Hungary; Department of Immunology and Biotechnology, Medical School, University of Pecs, Pécs, Hungary; Department of Immunology and Biotechnology, Medical School, University of Pecs, Pécs, Hungary; Department of Immunology and Biotechnology, Medical School, University of Pecs, Pécs, Hungary; Department of Pathology, Medical School, University of Pecs, Pécs, Hungary; Department of Pharmacology and Pharmacotherapy, Medical School, University of Pecs, Pécs, Hungary; Department of Immunology and Biotechnology, Medical School, University of Pecs, Pécs, Hungary; Department of Immunology and Biotechnology, Medical School, University of Pecs, Pécs, Hungary; Department of Immunology and Biotechnology, Medical School, University of Pecs, Pécs, Hungary

**Keywords:** autoimmune arthritis, splenectomy, cytokines, Treg

## Abstract

The spleen plays a role in innate and adaptive immunity, and autoimmune diseases like rheumatoid arthritis (RA). We investigated the effect of splenectomy in early and moderate stages of autoimmune arthritis in a mouse model. To induce recombinant human G1-induced arthritis (GIA), BALB/c mice were immunized intraperitoneally three times in 4-week intervals with the rhG1 antigen. Mice were splenectomized on day 7 (SPE1) or day 35 (SPE2) after the initiation of immunization; tested for clinical severity, joint radiological and histological changes, serum levels of inflammatory cytokines and autoantibodies, and rhG1-specific immune responses; and compared to those in control mice with spleen left intact. Circulating Tregs and T-helper subset ratios in the spleen and inguinal lymph nodes (LNs) were also examined using flow cytometry. The onset of severe inflammatory response was significantly delayed in SPE1 and SPE2 groups compared to control mice at early stages of GIA, which was associated with increased circulating Tregs. After the third immunization, as disease progressed, the severity scores were robustly increased in all mice. Nevertheless, in splenectomized mice, we observed reduced joint deterioration and cartilage damage, more Th2 cells in LNs, and reduced levels of pro-inflammatory cytokines and autoantibodies in their sera. Mesenteric LN cells of splenectomized mice exhibited weaker response *in vitro* against the rhG1 antigen compared to control mice spleen. In conclusion, splenectomy in the early stages of GIA delayed the inflammatory response, suggesting a protective effect against the development and progression of severe destructive arthritis.

## Teaser

We propose that splenectomy in early stages of autoimmune arthritis could be beneficial because it prevents bone and cartilage destruction in the joints. This might be mediated through increasing the circulating Treg frequency and decreasing the Th1/Th17 polarization which could contribute to reduced serum levels of pro-inflammatory cytokines and pathological autoantibodies.

## Introduction

Rheumatoid arthritis (RA) is a chronic debilitating systemic autoimmune disease, which is primarily manifested in the joints [1, 2]. RA manifests in chronic synovial inflammation and bone and cartilage destruction, and eventually leads to the complete loss of the joints’ function [2], affecting significantly the patients’ quality of life and causing an economic burden [3, 4]. Currently, there is no cure for RA; nonetheless, all available pharmacological therapies are restricted to slowing the progression of the joints’ deterioration and reducing extra-articular complications [1, 2, 5–7].

Mouse models of autoimmune arthritis, for example, collagen-induced arthritis (CIA), and proteoglycan-induced arthritis (PGIA) and its refined version, the recombinant human G1 (rhG1)-induced arthritis model (GIA) [8], have broadened our knowledge of RA pathophysiology and also allowed to evaluate potential anti-arthritic treatments and therapeutic strategies [9, 10]. Importantly, the GIA model closely resembles the human RA in its clinical and immunological aspects [8,11,12].

For mimicking all stages of human RA, mice in the GIA model are immunized three times at 3- to 4-week intervals with the rhG1 antigen intraperitoneally. Subsequently, the autoimmune arthritis develops through three phases. During the first phase, after the first immunization, pathological autoantibodies (rheumatoid factor—RF and anti-cyclic citrullinated peptide antibodies—anti-CCP) can already be detected in the serum before the appearance of clinical symptoms [8]. After that, mild undulating symptoms of inflammation start to develop, resembling the preclinical phase of human RA [8, 13, 14]. In the second phase usually manifested after the second immunization, most mice develop moderate to severe joint inflammation, corresponding to the early phase of clinical RA. After the third immunization, during the third phase of inflammation, mice develop severe clinical symptoms including irreversible limb deformities, pannus formation, substantial bone and cartilage damage, and eventually leading to the ankylosis of the joints [8, 13–15].

In addition to serving as a major secondary lymphoid organ, involved in B-cell differentiation and tolerance, hosting memory B cells, and also supporting peritoneal B-cell persistence [16–18], the spleen is also involved in the development of RA and other autoimmune diseases like systemic lupus erythematosus (SLE) and systemic sclerosis [19–22]. In our previous study [14], we reported that splenectomy 4 weeks prior to autoimmune arthritis induction did not alter the clinical picture of GIA [14]. Splenectomized mice developed RA similar to those of the control (spleen-preserved) group, despite the significant differences in circulating lymphocyte composition and serum parameters [14] based on which we concluded that the splenectomy led to a complex reorganization of the immune system and other secondary lymphoid organs might have compensated for the absence of the spleen [14].

Now we wanted to investigate what if the spleen is removed during the induction period of arthritis in the GIA model, which would correspond to the early phase of human RA. Therefore, BALB/c mice were splenectomized at different time points during immunization with the rhG1 antigen. We immunized all experimental groups three times intraperitoneally (i.p.) on days 0, 28, and 56, during which mice were randomized into three groups: (i) in group one, the spleen was preserved until the termination of the study (control group); (ii) group two was splenectomized on day 7 (SPE1); and finally, (iii) group three was splenectomized on day 35 (SPE2). We monitored the clinical parameters, joints’ histological and radiological changes, and serum parameters of all groups. Our findings demonstrate that splenectomy in early stages of RA induction mitigated the deterioration of the joints, associated with a decreased level of inflammatory cytokines and pathological autoantibodies in the serum. This indicates that splenectomy in early stages of RA may be an effective intervention to prevent the development of severe destructive arthritis.

## Materials and methods

### Mice

We used 4-month-old female BALB/c mice kept at the Department of Immunology and Biotechnology’s Mouse Facility under conventional conditions. All experimental protocols were approved by the Animal Welfare Committee of the University of Pécs (PTE-MÁB) and the experiments were conducted under the license number BA02/2000-13/2022 to F.B.

### Antibodies and reagents

All chemicals were purchased from Sigma–Aldrich unless otherwise stated. For ELISA, we used PBS containing 1.5% nonfat dry milk or 1% bovine serum albumin as blocking buffer, and PBS containing 0.5% or 0.05% Tween-20 as washing buffer for autoantibodies or cytokines ELISA, respectively. The following monoclonal antibodies were used for flow cytometry: anti-CD4-FITC and anti-CD25-APC from BD Biosciences (San Jose, CA, USA); anti-CD4-APC, anti-FoxP3-PE, anti-T-bet-PE-Cy7, anti-RORγt-PE, and anti-GATA-3-A488 all from Invitrogen (Carlsbad, CA, USA). For intracellular staining in flow cytometry, eBioscience Foxp3/Transcription Factor Staining Buffer kit (Carlsbad, CA, USA) was used.

### Induction and assessment of recombinant human G1-induced arthritis

Mice were immunized i.p. three times on days 0, 28, and 56 using the rhG1 antigen (40 μg) mixed with dimethyl-dioctadecyl-ammonium (DDA) adjuvant dissolved in PBS as described [14]. Arthritis severity and clinical signs were examined throughout the whole experiment every other day using a clinical scoring system as described [8, 14]. The diameter of mice paw thickness was measured using a digital caliper. E.K. was aware of the group allocation during the whole experiment. Confounders were not controlled. Mice were sacrificed 3 weeks after the third immunization (day 77) by cervical dislocation.

### Surgical procedure

Mice were randomized on an *ad hoc* basis into three groups: (i) spleen-preserved control (*n* = 9); (ii) group splenectomized on day 7 (1 week after the first immunization) annotated as ‘SPE1’ (*n* = 12); (iii) group splenectomized on day 35 (after the second immunization) annotated as SPE2 (*n* = 12). We anesthetized the mice using 100 mg/kg ketamine (Calypsol, Gedeon Richter, Budapest, Hungary) and 5 mg/kg xylazine (Sedaxylan, Eurovet Animal Health, Bladel, the Netherlands) i.p. before the operation. Mice were splenectomized as described [14]. The number of mice/group was determined based on previous studies.

### Micro-computed tomography

Mice were anesthetized (see above) and the ankle and foot structure was scanned using a SkyScan 1176 *in vivo* micro-computed tomography (micro-CT) system (Bruker, Kontich, Belgium). The scans were 3D reconstructed with CT Analyzer software. The anatomical nomenclature was utilized as described [23].

### Histological analysis

Arthritic mice (selected from each experimental group based on similar arthritis score) were sacrificed, and their hind limbs were collected and processed using standard histological procedures. Sections were stained with Mayer’s hematoxylin and eosin (H&E) and scanned using Pannoramic MIDI Scanner (3DHistech, Hungary). Images were analyzed using the Pannoramic Viewer Software (3DHistech).

### Sample collection and division

For the follow-up of the Tregs during the immunization protocol, we collected heparinized blood on days 25, 53, and 75 (before the second or third immunization or at the sacrifice, respectively) from five mice in all three experimental groups. At the end of the experiments, we collected the spleens from the control group and the mesenteric (mLNs) and inguinal LNs (iLNs) from all groups. In case of the LNs, due to the limited number of cells, we divided the samples as follows. We usually isolate three to four mLNs, which were divided for *in vitro* restimulation with the antigen for cytokine production or histology. In case of the iLN, one was used for flow cytometric analysis and the contralateral was used for histology.

### Flow cytometry

Blood and lymphoid organs were analyzed by flow cytometry. One million cells/sample were used and stained with different fluorochrome-conjugated anti-mouse monoclonal antibodies as described [24]. Data acquisition was performed using a FACS Canto II flow cytometer and for data analysis, FlowJo software v10 (BD Biosciences) was used. We defined the following cell subsets based on surface and intracellular markers: CD4^+^, T-helper cells; CD4^+^FoxP3^+^CD25^+^, regulatory T cells; CD4^+^T-bet^+^, T-helper-1 cells; CD4^+^T-GATA-3^+^, T-helper-2 cells; CD4^+^RORγ^+^, T-helper-17 cells.

### *In vitro* cell culture

Cells were isolated from the spleen of the control and mLNs of all mouse groups, and 1.8 × 10^6^ cells/well were cultured in DMEM supplemented with 10% fetal calf serum on a 48-well plate in the presence or absence of rhG1 antigen for 5 days.

### Cytokine and antibody ELISA measurements

We measured the IL-1β, IL-4, IL-6, IL-17, IL-23, IFNγ, and TNFα cytokine levels from the supernatants of the *in vitro* cell cultures and the sera using mouse ELISA kits from R&D Systems (Minneapolis, MN, USA), according to the manufacturer’s instructions. The serum levels of the rhG1 antigen-specific antibody and mouse proteoglycan-specific autoantibodies were measured using indirect ELISA as previously described [8, 14]. Levels of anti-CCP IgG1 and IgG2a antibodies in the serum were measured using the Immunoscan CCP Plus ELISA kit (SVAR, Malmö, Sweden) as previously described [14]. Serum levels of RF-IgG and RF-IgM were measured using the RF-IgG or RF-IgM FineTest Mouse ELISA Kits (FineTest, Wuhan, China) according to the manufacturer’s instructions.

### Statistical analysis

Data were analyzed using MS Excel. All data were presented as mean ± SEM. The experimental groups were compared using ANOVA with Tukey’s post hoc test, *P*-values ≤0.05 were considered statistically significant.

## Results

### Splenectomy during the induction of autoimmune arthritis delayed joint inflammation

**Figure 1.**
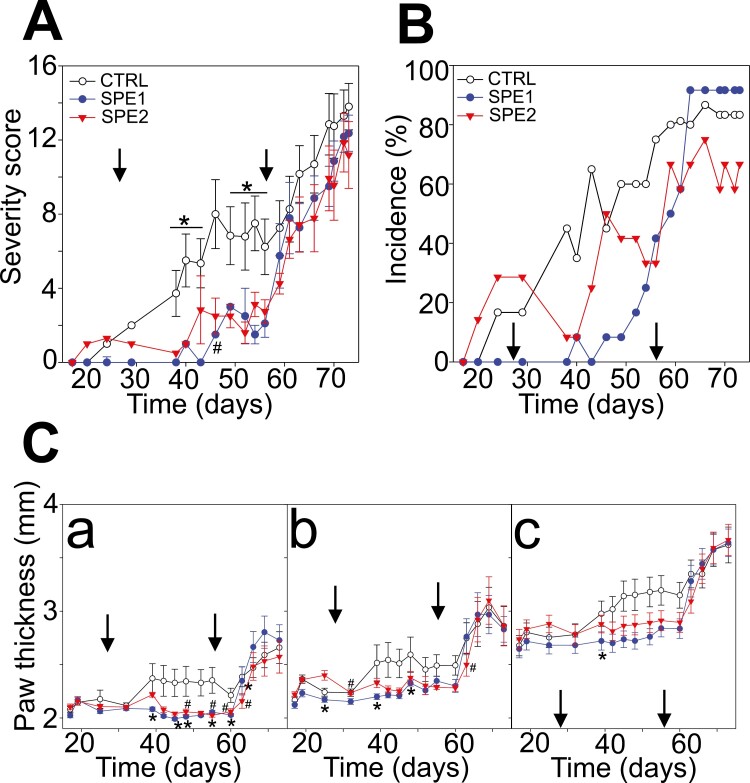
Development of autoimmune arthritis in splenectomized and control groups of mice using the GIA model. All mouse experimental groups were immunized with the rhG1 antigen three times on days 0, 28, and 56 (black arrows show the time of the second and third immunizations). Diagrams show the clinical severity score (**A**), incidence (**B**), and paw thickness (**C**) of spleen-preserved control (open circles, *n* = 12); splenectomized on day 7 (SPE1; filled circles, *n* = 12); and splenectomized on day 35 (SPE2; triangles, *n* = 12) groups. The paw thickness was measured in parallel with the scoring on (Ca) front paws, (Cb) hind paws, and (Cc) ankles in control (*n* = 5, open circles), SPE1 (*n* = 7, filled circles), and SPE2 (*n* = 6, triangles) mice. Results are presented as mean ± SEM. Statistically significant (*P* ≤ 0.05) differences are indicated as follows: SPE1(*) or SPE2(#) compared to the controls, respectively.

In the present experiment, we started the induction of GIA, and then groups of mice were either splenectomized 7 days after the first immunization (SPE1 group) or 7 days after the second immunization (SPE2 group), respectively, or spleen preserved (control group), and we followed the clinical picture (Fig. 1). The first signs of mild inflammation and swelling appeared ~20 days after the first immunization in the control and SPE2 groups; however, mice in the SPE1 group did not show any sign of inflammation during this period (before the second immunization; Fig. 1A–C). On day 28, we immunized all three mouse groups for the second time. As expected, we observed a significant and progressive increase in the severity scores of the control group, manifesting as moderate to severe redness and swelling (Fig. 1A and C). On the contrary, in the SPE2 group, splenectomized on day 35, the inflammatory signs of arthritis slightly decreased, and their clinical scores remained significantly lower than those of the control group until the third immunization (Fig. 1A). In addition, there was a transitional decrease in the incidence of the SPE2 group (Fig. 1B), until day 40; however, after that, their incidence soon reached a similar level to the control group (~50%) by day 45 (Fig. 1B). Surprisingly, the SPE1 group continued to remain resistant to inflammation; thus, their severity scores and incidence percentage persisted to be zero for approximately 2 weeks after the second immunization (day 42; Fig. 1A and B). Although on day 45 mild signs of inflammation started to appear, their severity scores remained significantly lower than those of the control group and slightly lower than the SPE2 group on days between 38 and 46 (Fig. 1A), respectively. Moreover, until day 56 (the time of the third injection), approximately 65% of the SPE1 mice did not develop any sign of arthritis (Fig. 1B).

To induce the severe stage and the irreversible form of autoimmune arthritis in the GIA model, we immunized the mice for the third time on day 56. We found a dramatic increase in severity scores and incidence percentages in all groups. However, the severity score of the control group persisted to be the highest until following the third immunization. At the end of the experiment, the control group average severity score was (13.8 ± 1.3), followed by SPE1 (12.4 ± 1) and SPE2 (11.2 ± 1.8; Fig. 1A). Furthermore, mice paw thickness values were eventually similar in all mouse groups (Fig. 1C). While approximately 90% of the mice in the SPE1 and control groups developed autoimmune arthritis the disease incidence was only 70% in the SPE2 group (Fig. 1B). These findings indicate that splenectomy can delay the progress of RA, although it cannot prevent its eventual worsening following repeated immunization.

### Splenectomy at early stage of autoimmune arthritis led to bone and cartilage preservation

**Figure 2.**
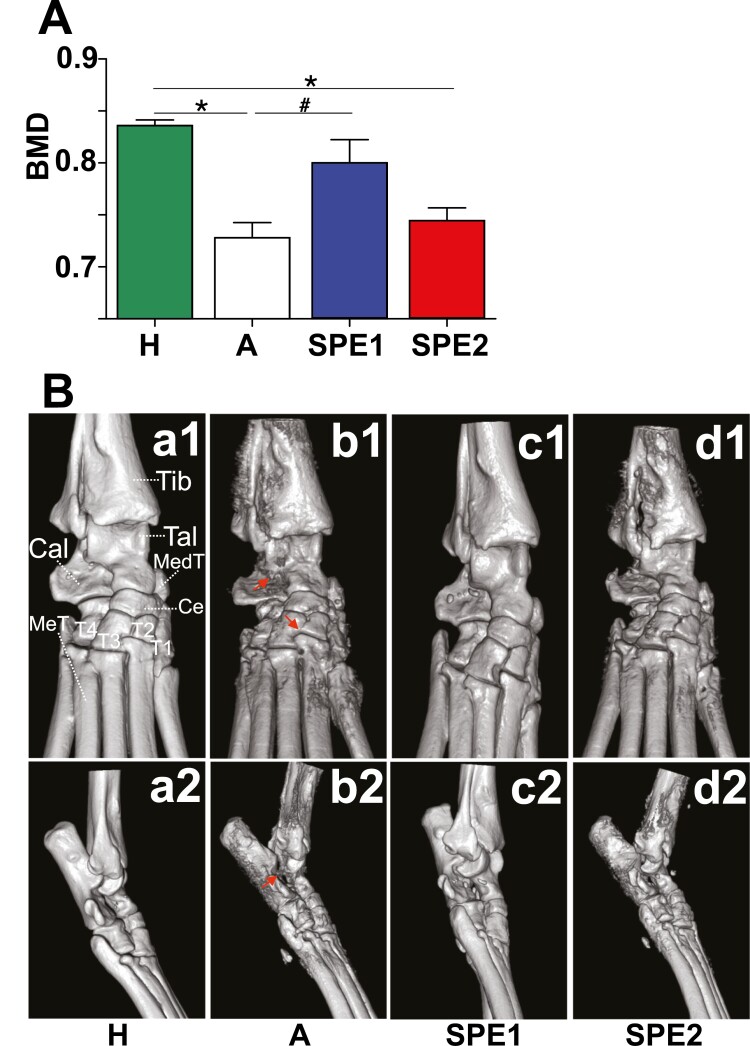
Micro-CT analysis of the ankle and proximal foot joints of healthy control and arthritic mice. (**A**) Analysis of the BMD measured in healthy (H) control (green, *n* = 3), arthritic (A) control (white, *n* = 4), SPE1 (blue, *n* = 4), and SPE2 (red, *n* = 4) groups. Bar diagrams show the mean ± SEM of the four experimental groups. Statistically significant (*^/#^*P* ≤ 0.05) differences are indicated compared to the healthy control or the arthritic control, respectively. (**B**) Representative images show the 3D microarchitectural changes of the tibial, tarsal, and metatarsal regions of the dorsal view (Ba1–d1) and lateral view (Ba2–d2) of healthy control (Ba1–2) or arthritic (Bb1–2) control, SPE1 (Bc1–2) and SPE2 (Bd1–2) mice, respectively. Red arrows indicate pathological bone adhesions in the arthritc control mice (Bb1 and Bb2). Abbreviations: Tib, tibia; Tal, talus; Cal, calcaneus; Ce, central tarsal bone; Med T, medial tibial bone; MeT, metatarsus; T1–4, tarsal bone I–IV.

To confirm our clinical findings, we performed micro-CT image analysis of the tibial, tarsal, and metatarsals (MeT) regions in the control, SPE1, and SPE2 groups to investigate the extent of the anatomical changes, bone deterioration, and damage of the affected joints (Fig. 2). As expected, the bone mean density (BMD) was significantly decreased in the arthritic (A) control group when compared to healthy (H) controls (Fig. 2A). The SPE1 mice had significantly higher BMD values compared to the arthritic controls, indicating less bone resorption (Fig. 2A). The BMD values of SPE2 mice were between the controls and the SPE1 (Fig. 2A). In line with the clinical picture, the arthritic control group showed the most severe bone surface irregularities, and deteriorations in the tibia, calcaneus, talus, medial tibia, tarsal, and metatarsal bones (Fig. 2Bb1 and Bb2). Moreover, the micro-CT images revealed adhesions between the Cal and Tal bones, and T2 and T3 bones of the control group, indicating that severe ankylosis developed in these mice (Fig. 2Bb1 and Bb2). The SPE1 group showed minimal bone surface irregularities (Fig. 2Bc1 and Bc2) when compared to those of the arthritic control group (Fig. 2Bb1 and Bb2). On the other hand, in SPE2 mice micro-CT image analysis (Fig. 2Bd1 and Bd2) showed severe irregularities and deteriorations of the bones similar to those of the arthritic control group (Fig. 2Bb1 and Bb2), however, without bone adhesions.

**Figure 3.**
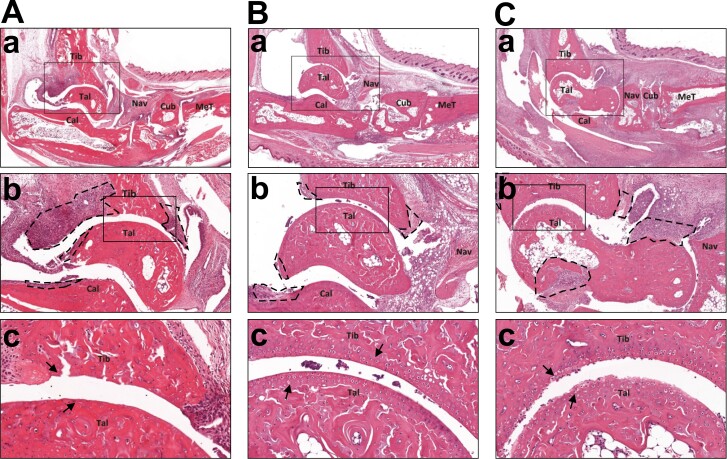
Histological analysis of the ankle joints and the proximal foot joints of splenectomized and control arthritic mice at the end of the experiments (stained with H&E). The panels show representative images from control (**Aa–c**), SPE1 (**Ba–c**), and SPE2 (**Ca–c**) with ×2 (panels ‘a’), ×8 (panels ‘b’), or ×25 (panels ‘c’) magnification, respectively. Rectangular areas show which regions were selected for higher magnification analysis. The synovial infiltrations and bone erosions are indicated as dashed line regions on the ‘b’ panels. Cartilage damage and degradation are indicated with black arrows on the ‘c’ panels. Abbreviations: Tib, tibia; Tal, talus; Cal, calcaneus; Nav, navicular bone; Cub, cuboid bone; MeT, metatarsus.

To further validate our results, next we assessed the inflammation and cartilage destruction of the ankle and small joints of the feet in histology (Fig. 3). H&E staining of the hind limbs revealed milder synovial inflammation, with less pannus formation and articular bone infiltration in splenectomized groups than in the control (Fig. 3Aa–Ca and Ab–Cb). Moreover, we found less articular cartilage damage in splenectomized mice than those in the control group (Fig. 3Ac–Cc). In fact, the cartilage surface on the tibia and talus of SPE1 group was preserved (Fig. 3Bc), unlike SPE2 where some cartilage erosion was observed (Fig. 3Cc); however, to a lesser extent than in the control group, where the cartilage was almost completely degraded (Fig. 3Ac). These results imply that splenectomy in the early stages of RA induction offered a protection against the destructive arthritis.

### Reduced levels of cytokines and autoantibodies in the sera of splenectomized mice with GIA

**Figure 4.**
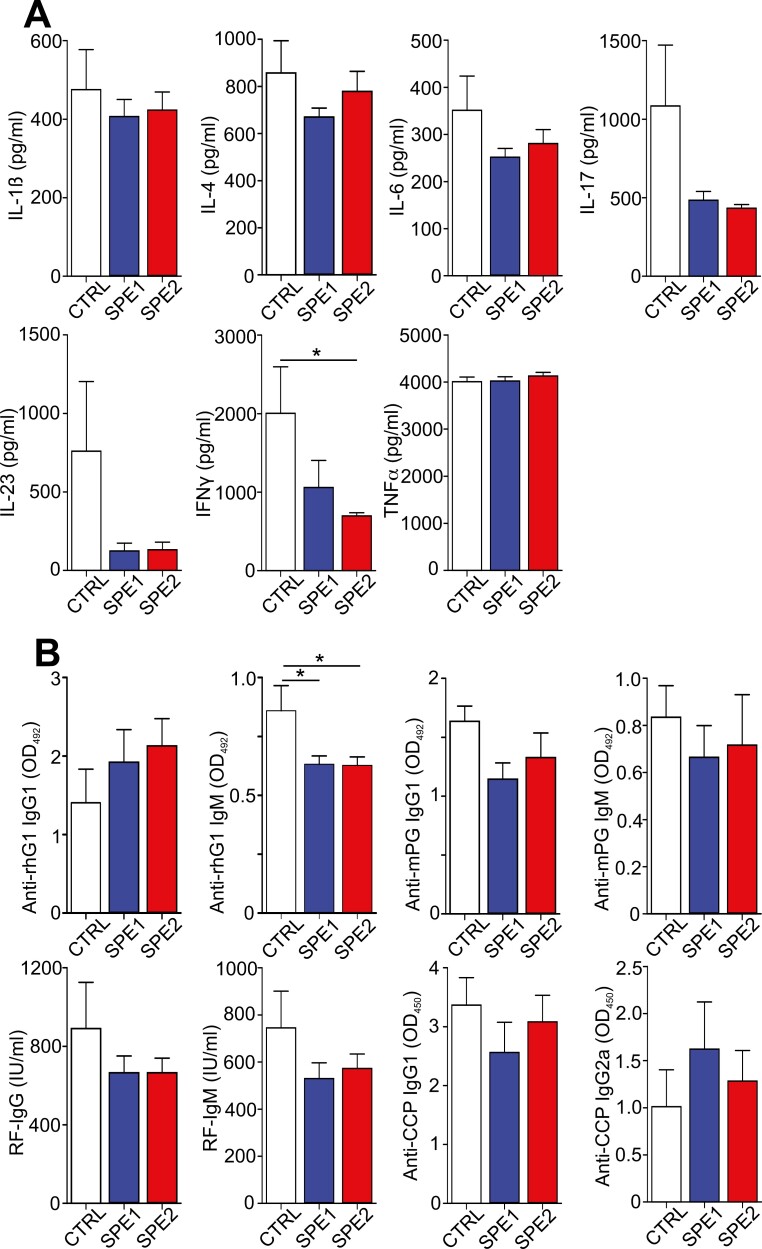
Analysis of the cytokine and antibody levels in the sera of the control and splenectomized arthritic mice. (**A**) Diagrams show the concentration of cytokines (pg/ml) in the sera of control (*n* = 9, white), SPE1 (*n* = 12, blue), and SPE2 (*n* = 12, red) mice. (**B**) Diagrams show the levels of anti-rhG1 IgG1/IgM (serum dilution 16 000×/100×, respectively), anti-mPG IgG1/IgM (serum dilution 100×), RF-IgG/IgM (serum dilution 100×/200×, respectively), and anti-CCP IgG1/IgG2a (serum dilution 50×) antibodies in the sera of control (*n* = 9, white bars), SPE1 (*n* = 12, blue bars), and SPE2 (*n* = 12, red bars) mice. Results are presented as mean ± SEM, statistically significant (*P ≤ 0.05) differences are indicated.

In the GIA model, a significant level of pro-inflammatory cytokines can be measured in the serum, and the immune response is accompanied with the production of pathological autoantibodies, including anti-mouse proteoglycan (anti-mPG), RF, and anti-CCP [8, 25–29]. Therefore, we wanted to investigate whether there was a correlation between the above-described clinical picture and the serum parameters. At the end of the experiment, we sacrificed the mice and collected their sera to measure the cytokine and autoantibody levels (Fig. 4). The serum level of the Th1/Th17 cytokines (IFNγ, IL-17, and IL-23) was the highest in the control group compared to SPE1 and SPE2 groups (although the differences were not statistically significant), together with the pro-inflammatory IL-1β and IL-6 (Fig. 4A), while the TNFα level was similarly high in all experimental groups (Fig. 4A). On the other hand, the level of anti-inflammatory Th2 cytokine (IL-4) was the lowest (not statistically significant) in the SPE1 group (Fig. 4A).

Among the serum antibodies, the highest level of anti-rhG1 IgG1 antibody was measured in the SPE2 group followed by SPE1, while the lowest was in the control (Fig. 4B). In contrast, SPE1 and SPE2 groups showed significantly lower levels of anti-rhG1 IgM than the control. Importantly, almost all types of autoantibodies tested (anti-mPG IgG1/IgM, RF-IgG/IgM, and anti-CCP IgG1) were lower (the differences were not statistically significant) in the splenectomized groups when compared to the control arthritic group (Fig. 4B). Only the anti-CCP IgG2a level was increased (not statistically significant difference) in SPE1 compared to the control and SPE2 groups (Fig. 4B). Taken together, splenectomy during the induction of autoimmune arthritis resulted in decreased autoantibody levels in the serum, which corresponded to the milder bone and cartilage deteriorations.

### Splenectomy altered *in vitro* cytokine production and skewed T-helper cell polarization in GIA

**Figure 5.**
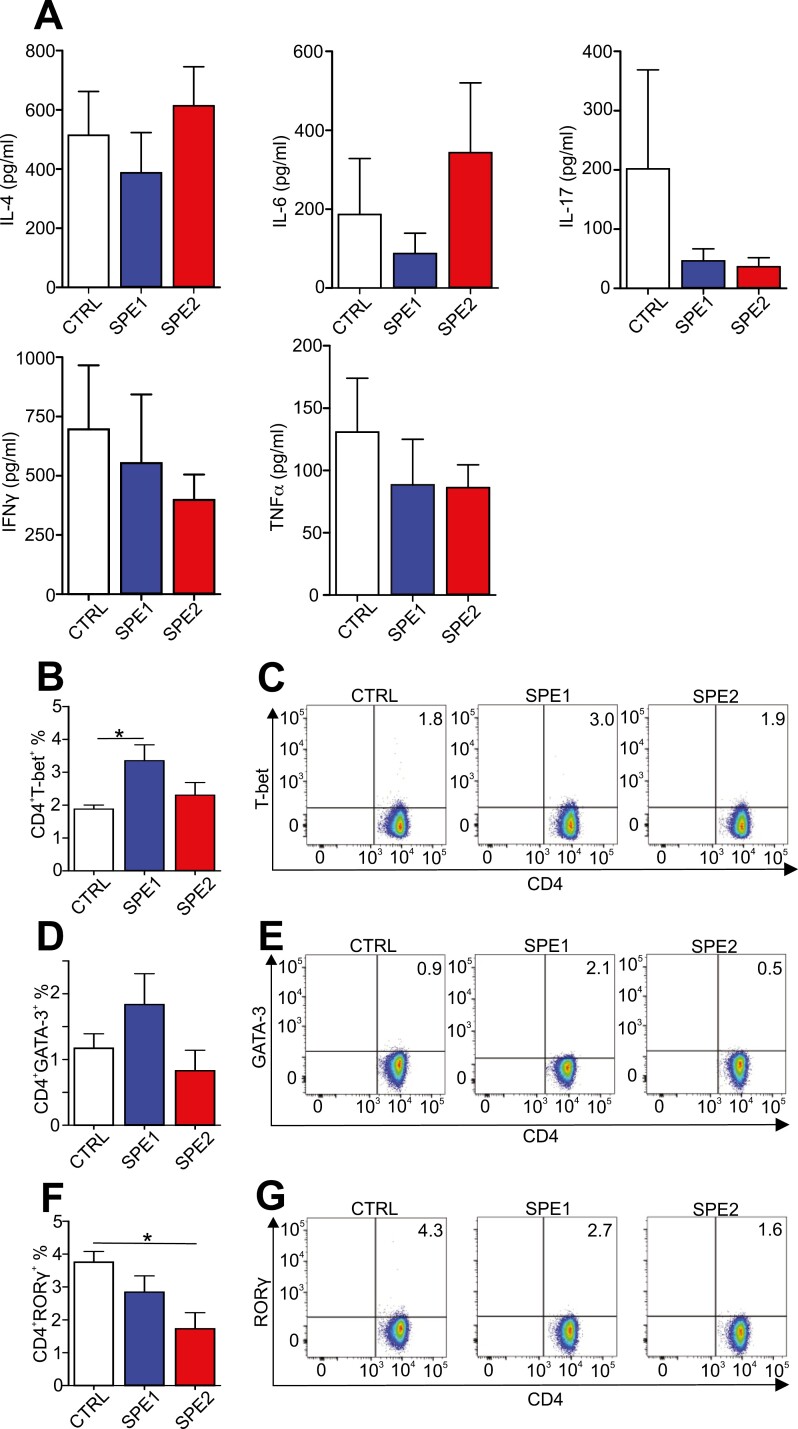
Comparison of the cytokine production and T-helper cell subsets in LNs of the control, SPE1, and SPE2 mice. (**A**) Diagrams show cytokine concentrations (pg/ml) measured using ELISA from supernatants of the *in vitro* rhG1-stimulated mLN cells of the control (*n* = 9, white), SPE1 (*n* = 12, blue), and SPE2 (*n* = 12, red) groups. (**B, D, F**) Diagrams show the ratios of Th1, Th2, and Th17 cell populations, respectively, isolated from iLNs of the control (*n* = 4, white), SPE1 (*n* = 5, blue), and SPE2 (*n* = 5, red) groups assessed by flow cytometry. Results are presented as mean ± SEM, statistically significant (*P ≤ 0.05) differences are indicated. (**C, E, G**) Representative flow cytometry density plots show percentages of Th1, Th2, and Th17 cells in the iLNs of all groups based on their CD4 and T-bet/GATA-3/RORγ staining, respectively.

Next, we investigated how the splenectomy affected the cellular immune response in autoimmune arthritis, so we measured the cytokines produced in response to rhG1 antigen in the supernatants of *in vitro* cultured mLN cells of all mouse groups (Fig. 5A). As severe arthritis also developed in both splenectomized groups, we hypothesized that, in the absence of the spleen, cellular immune responses also occurred in other secondary lymphatic organs. Therefore, we measured the cytokines produced by mLN cells of the splenectomized groups and compared them to those of the control group (Fig. 5A) similar to our previous study [14]. There was a decreased (but not statistically significant) IL-17, IFNγ, and TNFα production by the mLN cells of the splenectomized groups compared to the controls (Fig. 5A). As a reference, we also measured the cytokine production of the *in vitro* cultured spleen cells from the control arthritic group (Supplementary Fig. S1A). As expected, the spleen cells produced large quantities of IL-4 and IFNγ together with IL-6, IL-17, and TNFα (Supplementary Fig. S1A) which was in line with the cytokine composition observed in numerous previous studies [8, 24, 30, 31]. In general, the stimulation with rhG1 antigen elicited a stronger cytokine production in the spleen than the mLNs in any mouse groups (Supplementary Fig. S1A vs. Fig. 5A). These data implied that the cellular immune response during the autoimmune arthritis induction was predominant in the spleen, and its surgical removal during the induction (early phase) of autoimmune arthritis did not result in a compensatory immune response in the mLNs.

In the GIA model, we immunize BALB/c mice with the rhG1 antigen in combination with the DDA adjuvant causing a shift of the immune response toward CD4^+^-Th1 and CD4^+^-Th17, which is critical for the development of autoimmune arthritis [24, 30–32]. Here, we wanted to examine whether splenectomy during the induction of GIA influenced the T-helper cell polarization; thus, affecting the severity and clinical picture of the disease. Accordingly, we compared the frequencies of Th1, Th2, and Th17 in the iLN of the control and splenectomized groups (Fig. 5B–G). First, we found that the Th1 frequency was significantly higher in the iLNs of the SPE1 group, but only marginally higher in the SPE2 group than the control (Fig. 5B and C). Next, the Th2 frequency was the highest in iLNs of the SPE1 mice, while the lowest was observed in SPE2 mice compared to the control, with no statistical differences (Fig. 5D and E). Finally, the Th17 ratio was lower in the iLNs of both the SPE1 and SPE2 groups (only the latter being statistically significant) when compared to the iLN of the control group (Fig. 5F and G). In the spleen from the control mice, as expected [24, 30–32], we found predominantly Th1 and Th17 cells (Supplementary Fig. S1B and C). These observations support the notion that T-helper cell polarization was indeed influenced by the removal of the spleen in the early phase of GIA which might have, at least in part, contributed to the above-described differences in the clinical picture and serum parameters.

### Treg changes during the development of autoimmune arthritis in splenectomized mice

**Figure 6.**
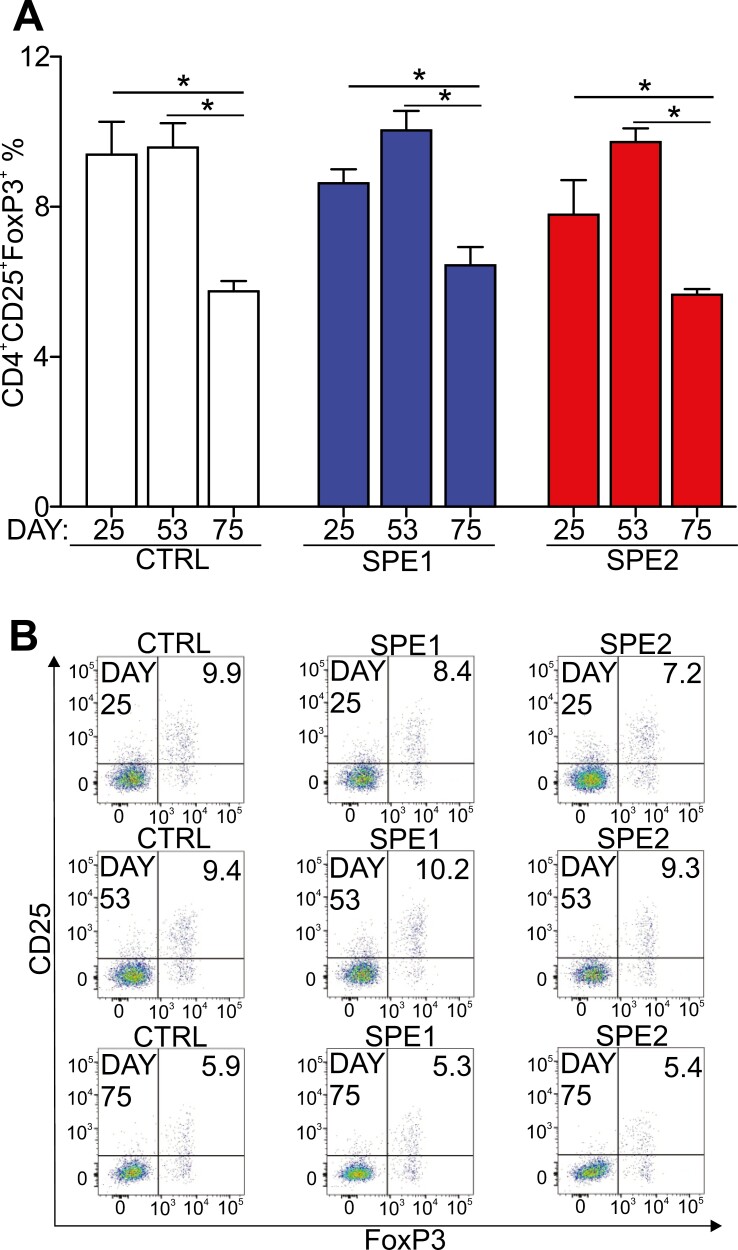
Comparison of the circulating regulatory T cells in control (*n* = 5, white), SPE1 (*n* = 5, blue), and SPE2 (*n* = 5, red) mice assessed by flow cytometry. (**A**) Diagrams show the ratio of Tregs (CD4^+^CD25^+^FoxP3^+^) at different time points during the induction of autoimmune arthritis. Results are presented as mean ± SEM, statistically significant (*P ≤ 0.05) differences are indicated. (**B**) Representative flow cytometry density plots show the characteristic distribution of circulating Tregs isolated from control, SPE1, and SPE2, based on their CD25 and FoxP3 staining. Numbers in the blots show percentages of the gated Tregs in the corresponding quadrants.

To explore the mechanisms underlying the decreased arthritis severity and joint damage observed in the splenectomized groups, finally we turned our attention to Tregs because they are essential in the regulation of autoimmune diseases including RA [2, 33–35]. We hypothesized that splenectomy during arthritis induction might have influenced the Treg frequency; we therefore assessed the circulating Tregs at different stages of arthritis development. In the control group, we observed that the percentage of Tregs did not change on days 25 and 53; however, after the third immunization (day 75), their percentage significantly declined (Fig. 6A and B). On the contrary, both splenectomized groups showed an increase (not statistically significant) in Treg percentages on day 53 (Fig. 6A and B) followed by a significant decline after the third immunization similar to the control group (Fig. 6A and B).

## Discussion

The spleen plays an essential role in both innate and adaptive immune responses especially in the protection against blood-borne antigens, and also for the removal of senescent and abnormal red blood cells and cellular debris [16, 36]. Additionally, the spleen is crucial for T-cell homeostasis and the development and maturation of B cells [36–39]. Enlargement of the spleen is common in autoimmune diseases including autoimmune hemolytic anemia, systemic sclerosis, SLE, and RA [19–22]. In our previous study [14], we have shown that splenectomy 4 weeks prior to first immunization to induce GIA did not prevent the development of autoimmune arthritis: the splenectomized group developed GIA with similar clinical picture as the spleen-reserved controls. Despite the similarity in the onset, severity, and incidence, we observed a significant shift in the immune response of the splenectomized group: we observed increased germinal center formation in the iLN and mLN upon the rhG1 immunization. Based on these results, we concluded that the removal of the spleen before the arthritis induction was compensated by other secondary lymphatic tissues (e.g. LNs) which manifested in a similar clinical picture to the arthritic spleen-preserved group [14].

In this study, we aimed to investigate the effect of splenectomy during the induction of GIA corresponding to the early and moderate stages of RA. Our results revealed that there was a significant delay in the inflammatory response in mice splenectomized during the immunization period with the rhG1 antigen. We divided our splenectomized mice into two groups: SPE1 and SPE2, splenectomized 1 week after the first or second immunization, respectively. The underlying rationale was that the GIA has three stages: after the first immunization, the autoimmune response starts corresponding to the preclinical stage of RA, which is followed by the second stage after the second immunization when the first clinical signs of arthritis develop corresponding to early and moderate stages of RA when the patients present with their first symptoms. Indeed, we saw important differences between the SPE1 and SPE2 groups: when we removed the spleen in the first phase (on day 7), the first signs of arthritis appeared only 2 weeks after the second immunization (between days 42 and 50). When we removed the spleen only after the second immunization (on day 35), when 30% of the mice had already mild signs of arthritis, there was a transient amelioration of the disease. Importantly, in either case, the inflammation of the limbs (severity scores and paw thickness) was milder than in the controls until the third immunization (day 56). To challenge this, we immunized all mouse groups for the third time to induce the severe, irreversible end-stage of autoimmune arthritis in GIA. In this last stage, the clinical severity scores were robustly increased in all three experimental groups; still, micro-CT images of the SPE1 group revealed profoundly less damage in their joints than arthritic control group. Moreover, SPE1 joints’ BMD was similar to those of the healthy mice, while the radiological changes of SPE2 mice showed a severe joint deterioration; however, it did not reach the level observed in the arthritic control group. These findings indicate that splenectomy in the early stage of RA ameliorated the development and progression of destructive arthritis. Histology also confirmed that there was less synovial inflammation, bone invasion, and cartilage destruction in the splenectomized mice, especially in the SPE1 group. We observed decreased pannus formation and bone erosions in splenectomized mice. As expected, the entire cartilage in the arthritic control was completely diminished, unlike in SPE2 mice, where we found localized cartilage erosions and irregularities, or SPE1, where most of the cartilage surface was intact.

The serological data were also in line with the clinical, radiological, and histological findings. There was a reduction in Th1 and Th17 signature cytokines (IFNγ, IL-17, and IL-23) in the SPE1 and SPE2 groups; however, the pro-inflammatory cytokines (IL-1, IL-6, and TNFα) showed similarly high levels in the sera of all experimental groups which could be due to the fact that the sera were collected only at the end of the experiment when all mice had already severe arthritis. Furthermore, we measured a reduced amount of RF, anti-CCP IgG1, and anti-mPG autoantibodies in the SPE1 and SPE2 groups than in the control group which, like in human RA, are relevant markers for the disease prognosis as correlate with the disease severity and level of the joints erosion [8, 26, 29]. The lack of significant differences between the autoantibody levels of the splenectomized and control mice could also be due to the fact that all sera were collected at the end of the experiments when all groups developed severe arthritis. In case of the anti-rhG1 antibodies, we measured similarly elevated IgG1 and lower IgM levels in the sera of splenectomized mice as in our previous work [14] corresponding to the earlier observations of Rozing et al. [40] about elevated IgG1 levels and decrease IgM after splenectomy.

We propose that splenectomy when performed 7 days after the first immunization (SPE1) led to the removal of activating B/T cells and memory precursors causing a very strong delay in the arthritis induction; whereas when the splenectomy was done 7 days after the second immunization (SPE2), when the rhG1-induced immune response was already established, it probably reduced the splenic resident memory B-cell subset without affecting the earlier differentiated and homed effector and memory cells (e.g. bone marrow-resident long-lived plasma cells or LN-resident memory cells).

To find further immunological details which could contribute to the above-described effects of splenectomy, we analyzed the frequency of the circulating Tregs, as they are essential in preventing and reducing the severity of RA [34, 35]. The increased frequencies of Tregs observed in the splenectomized mice in the early phase of GIA could explain the delay in the development of severe inflammation of the limbs. However, following the third immunization (late stage of GIA), the immunological tolerance was lost possibly due to the severe decline in the Treg frequency.

Additionally, in case of helper T-cell polarization, we found higher percentage of the Th1/Th17 lineage in the spleen of the arthritic control. In the iLNs of the SPE1, more Th2 cells were detectable, and less Th17 cells in both splenectomized groups compared to the control. So, the surgical removal of the spleen during the induction of autoimmune arthritis in GIA model resulted in the alteration of the T-helper cell polarization, too.

Taken together, we propose that splenectomy in the early stages of autoimmune arthritis could be beneficial because it prevents bone and cartilage destruction in the joints. This might be mediated through increasing the circulating Treg frequency and decreasing the Th1/Th17 polarization, which could contribute to reduced serum levels of pro-inflammatory cytokines and pathological autoantibodies.

## Supplementary Material

uxae013_suppl_Supplementary_Figures_S1

## Data Availability

The datasets used and/or analyzed during the current study are available from the corresponding author upon reasonable request.

## Abbreviations

CIA: collagen-induced arthritis
DDA: dimethyl-dioctadecyl-ammonium
FCS: fetal calf serum
GIA: G1-induced arthritis
iLNs: inguinal LN
PGIA: proteoglycan-induced arthritis
RA: rheumatoid arthritis
SLE: systemic lupus erythematosus
