# Supplementary figures and images for: Splenectomy at early stage of autoimmune arthritis delayed inflammatory response and reduced joint deterioration in mice
Source: Clin Exp Immunol. 2024 Feb 13;216(3):240–51. doi: 10.1093/cei/uxae013 (PMC11097914; doi:10.1093/cei/uxae013)

# Supplemental Figure 1

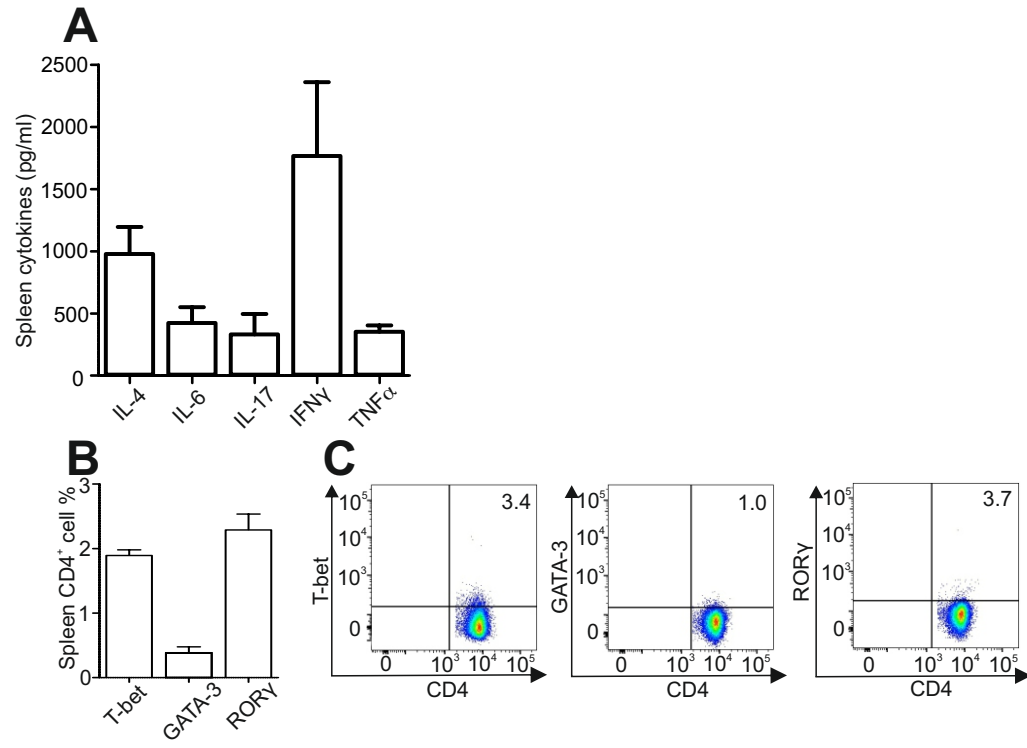

Supplement: uxae013_suppl_Supplementary_Figures_S1 [file uxae013_suppl_supplementary_figures_s1.pdf]
